# Nonsteroidal Anti‐Inflammatory Drugs as Modulators of Cation Channels: Fenamates Repurposing in Channelopathies

**DOI:** 10.1002/cmdc.202500301

**Published:** 2025-09-14

**Authors:** Paola Laghetti, Concetta Altamura, Simone Dell’Atti, Jean‐François Desaphy, Ilaria Saltarella

**Affiliations:** ^1^ Department of Precision and Regenerative Medicine Section of Pharmacology School of Medicine University of Bari Aldo Moro 70124 Bari Italy

**Keywords:** cationic channels, channelopathies, fenamates, Nonsteroidal Anti‐Inflammatory Drugs, pharmacology

## Abstract

Cationic ion channels are transmembrane proteins that regulate the flux of cations (potassium, sodium, and calcium) across cell membrane, playing a pivotal role in many cellular functions. Disruptions of their activity can lead to the so‐called genetic or acquired channelopathies, a heterogeneous group of diseases that affect multiple human systems. Fenamates, a class of nonsteroidal anti‐inflammatory drugs, has recently emerged as modulators of cationic ion channels highlighting the possibility of their repurposing for the treatment ion channel‐related disorders, such as channelopathies, chronic pain, epilepsy, cardiac arrhythmias, and cancers. In this review, the ability of fenamates (i.e. niflumic, flufenamic, mefenamic, meclofenamic, and tolfenamic acids) to differentially modulate the activity of cationic ion channels is described. Overall, preclinical and clinical studies suggest that fenamates represent a promising class of compounds for drug repurposing and for the development of new molecules, offering novel therapeutic opportunities for patients affected by ion channel‐related disorders.

## Graphical abstract

Beyond their established role as nonsteroidal anti‐inflammatory drugs (NSAIDs), fenamates (niflumic, flufenamic, mefenamic, meclofenamic, and tolfenamic acids) have recently been identified as modulators of cationic ion channels. This review highlights their differential effects on ion channel activity and explores their potential for repurposing in the treatment of channelopathies, chronic pain, epilepsy, cardiac arrhythmias, and cancer.

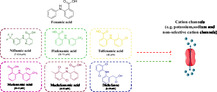

## 1 Introduction

Cationic ion channels are transmembrane proteins that control the flow of cations, including potassium (K^+^), sodium (Na^+^), and calcium (Ca^2+^), across cellular membranes. These proteins are pivotal in the maintainance of cellular homeostasis through the regulation of different cell functions, including neurotransmitter release, muscle contraction, signal transduction, hormone secretion, volume regulation, growth, and apoptosis.^[^ ^1^ ^]^

Disruptions in cation channel functions can lead to abnormal cellular signaling and dysfunction, leading to a variety of disorders, namely channelopathies. They represent a heterogeneous group of diseases that can affect the nervous, cardiovascular, respiratory, urinary, endocrine, and immune systems.^[^ ^2^, ^3^, ^4^ ^–^ ^6^ ^]^ Furthermore, emerging studies have investigated the involvement of ion channels in different solid and hematological cancers, including breast, ovarian, gastric, pancreatic, and multiple myeloma,^[^ ^7^, ^8^, ^9^, ^10^ ^–^ ^12^ ^]^ and also in neurodegenerative diseases or autoimmunity,^[^ ^13^ ^,^ ^14^ ^]^ leading to the definition of acquired channelopathies. These findings have further expanded the understanding of the pathological roles of ion channels, highlighting their relevance not only in genetic disorders but also in acquired conditions.

**Figure 1:**
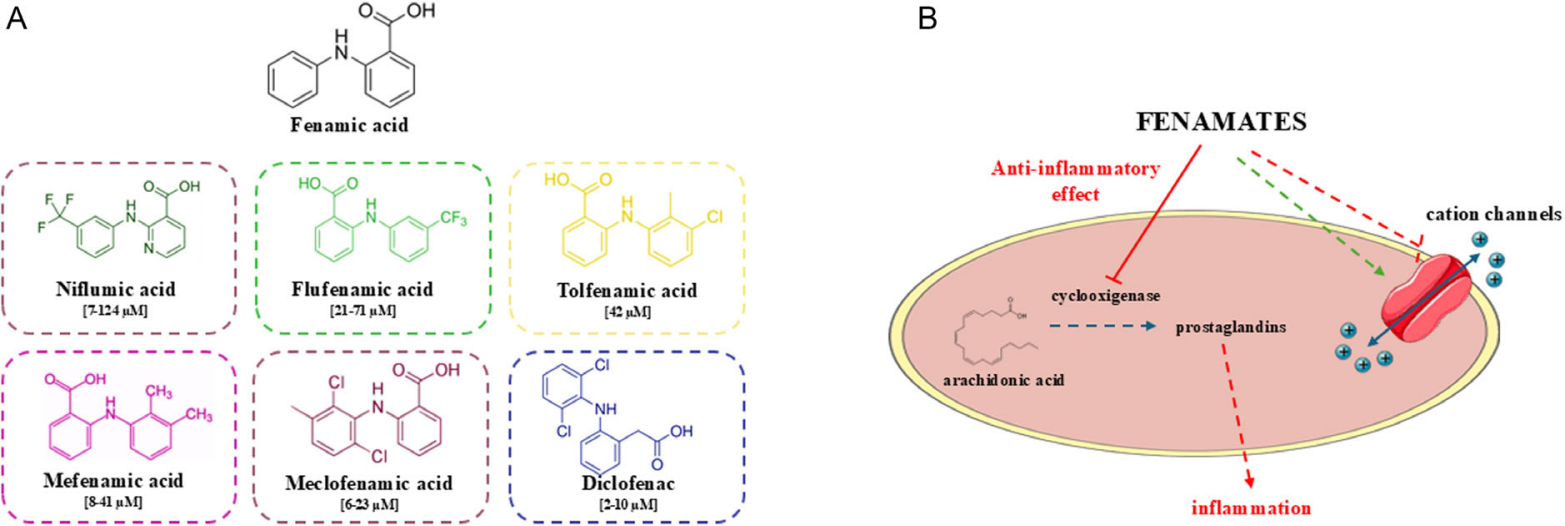
A) Structure of fenamic acid core and fenamates mentioned in the text. Plasmatic concentrations in human are reported in the square brackets.^[^ ^154^ ^]^ B) Effect of fenamates on the inflammatory cascade and ion channels.

Based on their pivotal role in many physiological and pathological cell functions, these channels represent attractive targets for the treatment of many systemic or multi‐systemic diseases (i.e., cardiac disorders, epilepsy, neuromuscular diseases, and cancers). Nevertheless, despite literature data highlights the involvement of ion channels in many disorders, less than 10% of currently marketed drugs directly target ion channels, suggesting their underestimated therapeutic potential as well the need to identify novel molecules able to modulate their activity.^[^ ^15^ ^]^ Among approved drugs, fenamates, a group of nonsteroidal anti‐inflammatory drugs (NSAIDs), have emerged for their ability to modulate ion channels. Fenamates are anthranilic acid derivatives that exert their anti‐inflammatory effect through the inhibition of cyclooxygenase enzymes and the following interference with the arachidonic pathway and prostaglandin synthesis. Fenamates currently used in clinical practise include niflumic acid (NFA), meclofenamic acid (MCFA), flufenamic acid (FFA), tolfenamic acid (TFA), and mefenamic acid (MFA), mainly prescribed to treat mild to moderate pain, rheumatoid disorders, migraine headaches, and idiopathic dysmenorrhea^[^ ^16^ ^,^ ^17^ ^]^ (**Figure** **1**). However, beyond their anti‐inflammatory activity, these drugs are acquiring increasing interest for their effects on ion channels opening new possibilities for their therapeutic use. Accordingly, previous studies revealed that fenamates display many additional pharmacological properties, including their ability to modulate cationic channels, with both activating and inhibitory effects.^[^ ^18^ ^]^ Despite the mechanisms involved in the modulation of ion channel activity still need to be further elucidated, fenamates could be an intriguing class of drugs that may serve as pharmacological tools to investigate the biological functions of ion channels and that could potentially be repurposed for treating primary or acquired ion channelopathies. The analysis of fenamates effects on different ion channel isoforms should provide a comprehensive overview of their therapeutic potential for the treatment of many diseases characterized by ion channel dysfunction. We have previously reviewed the modulatory effects of fenamates on chloride ion channels, highlighting their potential for drug repurposing in the treatment of chloride channel‐related channelopathies.^[^ ^19^ ^]^ Therefore, as cationic ion channels (e.g., potassium, sodium, and non‐selective cation channels) are involved in various diseases with limited therapeutic options, this review aims to summarize the latest findings on fenamates’ ability to modulate cationic ion channels activity with a particular focus on their potential therapeutic repurposing for the treatment of genetic and acquired channelopathies.

## 2 Potassium Channels and Fenamates

Potassium channels are expressed in both excitable and non‐excitable cells, and they are classified in three groups according to structure and function: voltage‐gated channels (Kv) (including calcium‐activated K^+^ channels (CaK)), tandem pore domain (K_2P_) channels, and inwardly rectifying potassium channel (Kir).^[^ ^20^ ^]^

The discovery of new molecules targeting potassium channels is of great interest because of their numerous roles in human cells, including the repolarization of excitable cells (e.g., muscle and neuronal cells after an action potential), the maintenance of electrolyte homeostasis in nephrons, the proliferation of T‐ and B‐cells, and the auditory signal transduction.^[^ ^21^ ^]^

**Figure 2:**
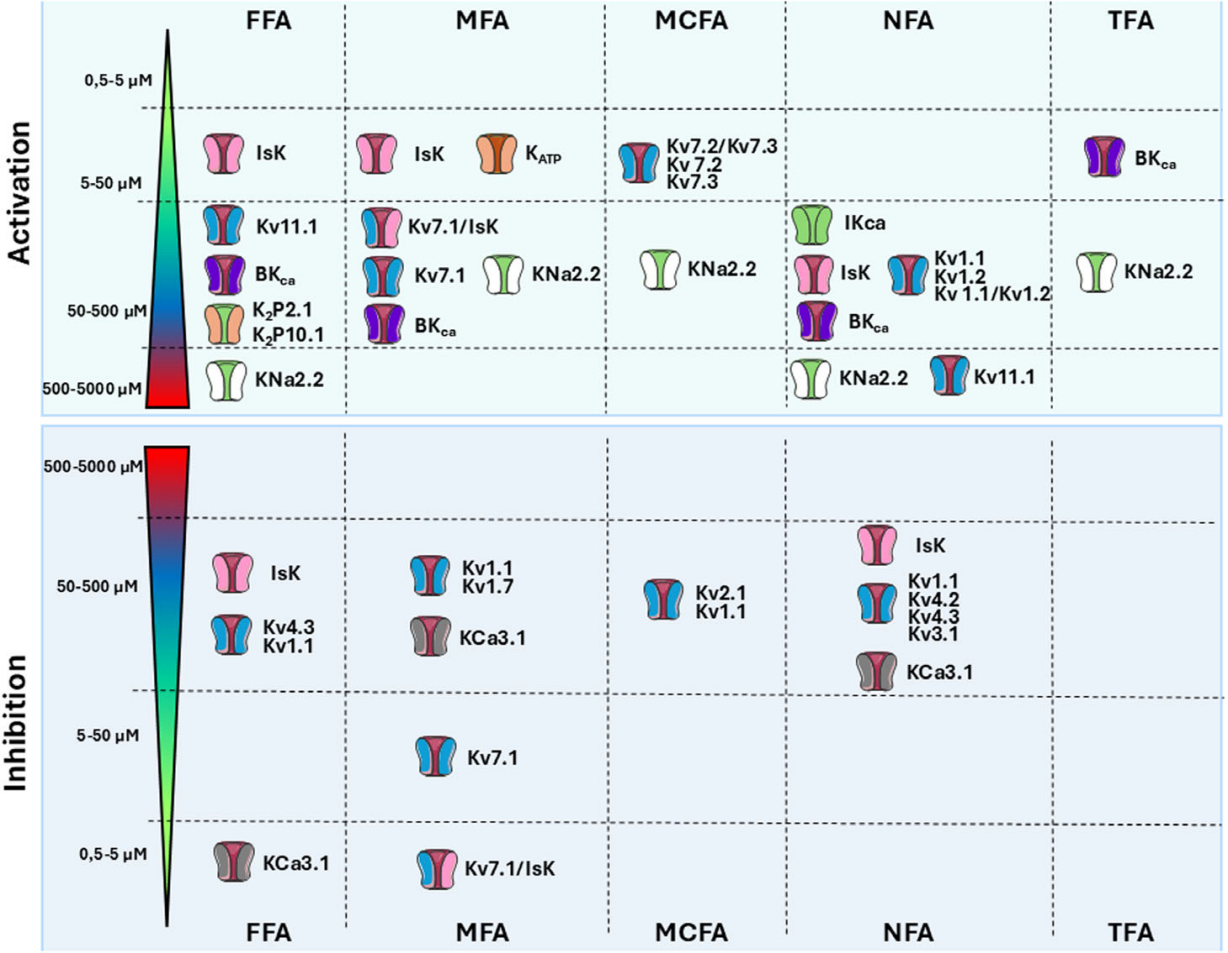
Effects of fenamates on potassium channels. Colored scale reported the estimated range of IC50 or concentration tested for each fenamates. These inhibitory or activation values are not always fully comparable since they have been evaluated on different systems (heterologous or native systems, in vitro or ex vivo) by considering different effects (current amplitude, voltage dependence). Dual effects of MFA and FFA are reported on IsK, Kv7. 1 and IsK/Kv7. 1channels. Detailed information, abbreviations, and references are provided within the text.

The potential activity of fenamates has been explored since the 1990's, when it was observed that MFA and FFA activated outward potassium currents in epithelium and smooth muscle cells^[^ ^22^, ^23^, ^24^ ^–^ ^25^ ^]^ (**Figure** **2**).

### 2.1 Voltage‐Gated Potassium Channel (Kv)

The voltage‐dependent potassium channel (Kv) family can be divided into nine subfamilies, from Kv1 to Kv9, consisting in pore‐forming alpha subunits associated with auxiliary beta subunits.

In cultured rat cerebellar granule neurons, FFA has a dual effect on the voltage‐activated transient outward K^+^ current (I_A_); low FFA concentrations [0.1–10 µM] increased I_A_, whereas high FFA concentrations [20 µM–1 mM] reversibly reduced current amplitude.^[^ ^26^ ^]^ However, the effects appeared independent on Kv1.1 (KCNA1 gene), Kv4.2 (KCND2), and Kv4.3 (KCND3) channels, which are thought to contribute to I_A_. It was also suggested that FFA may interact with both intracellular and extracellular sides of the channel.^[^ ^26^ ^]^ Similar effects were observed with MFA, that is, I_A_ activation at 1 µM and reversible I_A_ inhibition at 5–100 µM.^[^ ^27^ ^]^

The Kv2.1 channel (KCNB1 gene) carries delayed‐rectifier K^+^ currents in neurons, which gain‐of‐function missense variants are associated with epileptic encephalopathy.^[^ ^28^ ^]^ Lee and collaborators tested MCFA, MFA, FFA, and NFA on CHO cells transfected with the human isoform of this channel and demonstrated that 100 µM MCFA reversibly blocked Kv2.1 current in a dose‐dependent and voltage‐independent way without affecting the activation and deactivation speed. Conversely, other fenamates had weak or null effect on current amplitude.^[^ ^29^ ^]^

Compared to hKv2.1, inhibitory effects of MCFA were less pronounced on hKv1.1 (KCNA1 gene), while those of NFA and FFA were greater; MFA had no effect on both channels. However, these results were recently challenged by Servettini and collaborators, who showed enhancement of Kv1.1 channel voltage‐dependent activation by NFA in transfected Xenopus oocytes and HEK293 cells, as well as in the neuro‐2a cell line.^[^ ^30^ ^]^ NFA also enhanced K^+^ currents carried by homomeric Kv1.2 or heteromeric Kv1.1/Kv1.2 in HEK293 cells. Interestingly, NFA enhanced the Kv1.1 variant p.V408A expressed in Xenopus oocytes, which is associated with human episodic ataxia type 1 (EA1). The drug was able to restore amplitude and frequency of inhibitory post‐synaptic currents, as well as firing frequency, in cerebellar Purkinje cells from Kv1.1^V408A/+^ mice. According to the authors, such an effect may result from Kv1.1 and Kv1.2 channels enhancement in presynaptic basket cells terminals, which may reduce GABA release acting on postsynaptic Purkinje cells.^[^ ^30^ ^]^ Yet, a direct effect of NFA on GABA_A_ receptor cannot be excluded [see above]. In vivo, NFA administration to Kv1.1^V408A/+^ mice ameliorated the motor performance, suggesting a potential use in EA1 patients. In addition, NFA was shown to enhance the Shaker K^+^ channel, the fly Kv1.1 ortholog, expressed in *Xenopus* oocytes. Such effect likely contributes to the improvement of motor performance and neuromuscular transmission in *Drosofila* flies carrying a Shaker missense mutation associated with an EA1‐like phenotype.^[^ ^30^ ^]^

The Kv3.1 channel (KCNC1 gene) is mostly found in excitable cells,^[^ ^31^ ^]^ being involved in neuronal excitability,^[^ ^30^ ^]^ proliferation and differentiation of neuron precursors,^[^ ^32^ ^]^ and muscle contraction.^[^ ^33^ ^]^ Reduced Kv3.1 function has been linked to Alzheimer's disease,^[^ ^34^ ^]^ inherited ataxia,^[^ ^35^ ^]^ schizophrenia,^[^ ^36^ ^]^ cognitive impairment, ^[^ ^37^ ^]^ and progressive myoclonus epilepsy.^[^ ^38^ ^]^ Costa and colleagues showed that, in HEK293 cells, NFA inhibits both endogenous swelling‐activated Cl^−^ currents and Kv3.1‐related K^+^ currents. As NFA concentrations able to reduce Kv3.1 activity fall within the NFA plasma values after oral administration, the authors suggested that the use of NFA may be unsafe in patients with Kv3.1 linked diseases.^[^ ^39^ ^]^

The Kv7 family is encoded by KCNQ genes and includes Kv7.1 (KCNQ1), Kv7.2 (KCNQ2), Kv7.3 (KCNQ3), Kv7.4 (KCNQ4), and Kv7.5 (KCNQ5) channels. In neurons, the heteromeric Kv7.2/7.3 channel is the molecular correlate of the M‐type K^+^ currents, which is critical to hamper neuron excitability. The loss of function of KCNQ2 and KCNQ3 variants is associated with human epileptic syndromes. Peretz and collaborators showed that MCFA acts as an opener of heteromeric Kv7.2/7.3 channels expressed in CHO cells or *Xenopus* oocytes, increasing current amplitude by inducing a hyperpolarizing shift of the activation voltage dependence and slowing of deactivation.^[^ ^40^ ^]^ Drug effect was more accentuated on Kv7.2 compared to Kv7.3 homomeric channels. No significant effect on current amplitude was observed on other potassium channels like Kv1.2 (KCNA1), Kv1.5 (KCNA5), Kv2.1 (KCNB1), and Kv7.1 (KCNQ1). Importantly, MCFA increased M‐type K^+^ currents and reduced spontaneous and evoked spiking discharges in cultured rat cortical neurons; in vivo, MCFA exerted anticonvulsant effects in the maximal electroshock seizure (MES) test in mice, when administered intraperitoneally at 50–100 mg kg^−1^, but was proconvulsive at the dose of 200 mg kg^−1^.^[^ ^40^ ^]^ These data suggested that MCFA could be used as a template for the development of new drugs targeting KCNQ2 and KCNQ3. A series of MCFA derivatives were developed, allowing to define pharmacophoric features of M‐type channels for openers and blockers as well as crucial determinants for cyclooxygenase inhibition. Openers of heterologously expressed Kv7.2/7.3 channels were found with IC_50_ values as low as a few µM in transfected CHO cells, which displayed potent firing inhibition in rat hippocampal and dorsal root ganglion (DRG) neurons and anticonvulsant effects in the MES mouse model.^[^ ^41^ ^]^ Recent data suggest that MCFA may have additional and/or synergistic effects with other known Kv openers, such as retigabine and diclofenac, in increasing M‐type K^+^ currents and reducing excitability of rat DRG nociceptors. Accordingly, MCFA/regitabine reduced pain‐like behaviors in a rat model of Gulf War illness suggesting the improved analgesic potency of this pharmacological combination.^[^ ^42^ ^]^

Fenamates may also affect K^+^ currents involved in cardiac action potential. NFA shifts the voltage‐dependence of Kv4.2 and Kv4.3 channels expressed in Xenopus oocytes, which contribute to the transient outward current in cardiac myocytes.^[^ ^43^ ^]^ Maximal effect was greater on Kv4.3, resulting in significant reduction of current amplitude. This result raises questions about the frequent use of fenamates to discriminate between K^+^ and Cl^−^ currents contribution to cardiac action potential.

In the heart, Kv7.1 assembles with the auxiliary subunit IsK (KCNE1 gene) to form heteromeric potassium channels responsible for the cardiac slow delayed rectifier K^+^ current (I_Ks_), an attractive pharmacological target for cardiac arrhythmias treatment. NFA, MFA, and FFA positively regulated the K + currents recorded in *Xenopus* oocytes transfected with KCNE1, likely through stabilization of the channel in the open state.^[^ ^44^ ^]^ MFA effect likely depends on the interaction with IsK subunit, since the mammalian homomeric Kv7.1 was insensitive to the drug.^[^ ^45^ ^]^ MFA enhanced heteromeric Kv7.1/IsK channels in *Xenopus* oocytes and rescued K^+^ currents carried by heteromeric channels containing dominant‐negative KCNE1 variants, including a naturally occurring LQT5 one.^[^ ^46^ ^]^ In CHO‐K1 cells, MFA slightly reduced homomeric Kv7.1 currents but greatly increased heteromeric Kv7.1/IsK currents.^[^ ^47^ ^]^ The effects on I_Ks_ were dependent on the temperature of bath solutions used for patch clamp experiments. At 20 °C, MFA induced a switch from a slow activation kinetics to an instantaneous onset, with a linear I–V relationship that suggested a permanent activation of I_Ks_. When the temperature was increased to 37 °C, MFA still increased I_Ks_ but the activation kinetic returned to its basal condition, resulting in no change in the sigmoidal I–V curve and shifting the voltage activation to more hyperpolarized potential. Overall, these data suggested a role of IsK subunit in the regulation of pharmacological response and temperature sensitivity of the channel. Kv7.1/IsK channel activation by MFA was further confirmed in HEK293 cells, where MFA enhanced I_Ks_ in a dose‐ and channel subunit stoichiometry‐dependent way.^[^ ^48^ ^]^ Conversely, MFA inhibits both homomeric KCNQ1 and heteromeric KCNQ1/KCNE1 zebrafish isoforms expressed in CHO cells, suggesting differences between species.^[^ ^49^ ^]^

In cardiac myocytes, the rapidly activating delayed rectifier K^+^ current (I_Kr_), carried by Kv11.1 channels (hERG or KCNH2 gene), also contributes to action potential repolarization. Inhibition of Kv11.1 by pharmacological agents or by inherited mutations (LQT2) causes the prolongation of QT interval (Long QT, LQT), which predisposes to severe life‐threatening arrhythmias.^[^ ^50^ ^]^ FFA and NFA were shown to increase I_Kr_ in *Xenopus* oocytes transfected with KCNH2.^[^ ^51^ ^]^ The concurrent activation of I_Ks_ and I_Kr_ by fenamates suggests a potential antiarrhythmic effect of these drugs, especially for the prevention and treatment of LQT syndrome.

#### 2.1.1 Calcium‐Activated K^+^ Channels (CaK)

Calcium‐activated K^+^ channels can be distinguished in three main types, depending on their single channel conductance and pharmacological characteristics, including large‐conductance (BK_Ca_ or KCa1.1 encoded by *KCNMA1* gene), intermediate‐conductance (IK_Ca_ or KCa3.1, encoded by *KCNN4*), and small‐conductance (SK_Ca_ or KCa2.x encoded by KCNN1 to KCCN3 genes) Ca^2+^‐activated K^+^ channels.^[^ ^52^ ^]^ Closely related are K^+^ channels activated by Na^+^ ions (Slo2.2 or KNa1.1 channel encoded by *KCNT1* gene, Slo2.1 or KNa1.2 channel encoded by *KCNT1*), or high intracellular pH (Slo3 or KCa5.1 channel encoded by *KCNU1* gene).

The activation of BK_Ca_ by fenamates was early hypothesized in studies showing an increase in BK_Ca_ currents after application of NFA in various smooth muscle cells preparations.^[^ ^53^, ^54^ ^–^ ^55^ ^]^ The IC_50_ for NFA was 260 µM in pig coronary smooth muscle membrane vesicles.^[^ ^53^ ^]^ NFA exerted a leftward shift of both voltage and Ca^2+^‐dependent activation. FFA was as potent as NFA, while MFA was less potent, suggesting a role for the polar ‐CF_3_ group common to the former. The effects were more pronounced when the fenamates were applied externally and were reversible. These effects were confirmed on cloned mouse and human BK_Ca_ channels expressed in *Xenopus* oocytes and HEK cells.^[^ ^56^ ^]^ In pig urethra myocytes, MFA activated both ATP‐sensitive K^+^ (K_ATP_) channels and BK_Ca_ channels, while inhibition of spontaneous transient outward K^+^ channels was reported.^[^ ^57^ ^]^ In guinea pig vascular smooth muscle cells, NFA and FFA activated BK_Ca_ channels with an IC_50_ of ≈300 µM.^[^ ^58^ ^]^ Activation of BK_Ca_ channels likely contributes to muscle relaxation induced by fenamates in various preparations, including guinea pig trachea,^[^ ^59^ ^]^ cultured human and bovine eye trabecular meshwork,^[^ ^60^ ^]^ and cultured human airway smooth muscle cells.^[^ ^61^ ^]^ Interestingly, FFA reduced excessive airway constriction in a mouse model of asthma in vivo.^[^ ^61^ ^]^

Besides smooth muscle cells, NFA and MCFA were shown to increase BK_Ca_ activity in a human osteoblast‐like cell line with an IC_50_ of ≈25 µM.^[^ ^62^ ^]^ TFA and FFA were less efficient in activating BK_Ca_ channels. In osteoblasts, BK_Ca_ channels participate in modulating osteocalcin secretion and voltage gated Ca^2+^ and Na^+^ channels activity. In transgenic mice with conditional knockout of Slo1 in osteoblasts, the deficiency of BK_Ca_ reduced bone density by altering the Wnt/*β*‐catenin signaling pathway.^[^ ^63^ ^]^ These results suggest that fenamates might modulate osteoblast functionality with possible implication in osteoporosis treatment.

The effects of fenamates are not limited to BK_Ca_. NFA (100 µM) was also shown to activate native IK_Ca_ currents in isolated mouse aortic endothelial cells by shifting leftward the Ca^2+^‐dependent activation curve.^[^ ^64^ ^]^ However, in 3T3‐L1 fibroblasts, an embryonic mouse cell line, FFA was identified as a KCa3.1 inhibitor at low concentration (IC_50_ 1.6 µM), while NFA and MFA showed inhibitory effect at concentration higher than 50 µM.^[^ ^65^ ^]^

Regarding large K^+^ conductance activated by Na^+^, a study in *Xenopus* oocytes expressing Slo2.1 (KNa2.2) channels showed that fenamates activated the channel in the absence of intracellular Na^+^ and independently from transmembrane‐voltage, stabilizing its open state in a concentration‐dependent manner.^[^ ^66^ ^]^ These effects were observed with millimolar concentrations of FFA and NFA (IC_50_ = 1.6 and 2.4 mM, respectively). Further investigation reported a biphasic action with an early rapid activation phase followed by a partial inhibition of currents after prolonged exposure.^[^ ^67^ ^]^ The concentration‐response relationships indicated that MCFA was the most potent activator of Slo2.1 channels, followed by TFA > MFA > FFA > NFA. Yet, NFA showed the greatest efficacy suggesting the existence of separate binding sites differentially activated by fenamates. The point mutation Ala278Arg in the S6 segment increased spontaneous activity of Slo2.1 and activation by NFA, while reducing NFA inhibitory effect. In the same study, NFA and MCFA were shown to activate also Slo2.2 (KNa2.1), but with lower potency.

### 2.2 Tandem Pore Domain Potassium Channels

Tandem pore domain potassium channels (K_2P_) are responsible for the generation of the leak potassium currents that stabilize negative membrane potential counteracting membrane depolarization.^[^ ^68^ ^]^ The K_2P_ superfamily counts six subgroups including the TREK (TWIK‐Related K^+^ Channel) subfamily, which is sensitive to fenamates. TREK family includes TREK‐1 (K_2P_2.1), TREK‐2 (K_2P_10.1), and TRAAK (K_2P_4.1) channels, involved in stabilization of membrane potential and regulation of neuronal excitability. In addition, a number of transcriptional variants have been described with variable activity. FFA, NFA, and MFA activate TREK and TRAAK channels in a concentration‐dependent manner, with FFA resulting in the most potent drug (IC_50_ = ≈100 µM), especially on TREK1 and TREK2.^[^ ^69^ ^]^ A similar observation was made with BL‐1249, a fenamate analog not used in the clinics due to unfavorable pharmacokinetics.^[^ ^70^ ^,^ ^71^ ^]^ Interestingly, BL‐1249 was quite selective for TREK‐1 and TREK‐2 (IC_50_ = 5–10 µM) compared to all the other K2P channels. Thus, BL‐1249 was used recently as the starting compound for the development of new drugs with improved pharmacokinetics, which led to a new promising TREK‐1/2 dual activator, namely 17f.^[^ ^72^ ^]^ Concerning the longer and the shorter TREK‐1 transcriptional variants, Veale and colleagues observed that fenamates had lightly different effects based on TREK‐1 length.^[^ ^70^ ^]^ FFA, MFA, NFA, and BL‐1249 were shown to enhance current through the shorter TREK‐1 isoform revealing a selective K^+^ conductance. Based on the involvement of TREK‐1 in pain sensation, it was proposed that TREK‐1 enhancement by fenamates may contribute to the analgesic effect of these drugs.

TRESK (TWIK‐related spinal cord potassium channel/K_2P_18.1) is another channel belonging to K_2P_ potassium channel family, encoded by *KCNK18* gene and mainly expressed in peripheral sensory neurons, trigeminal neurons and dorsal root ganglia. The opening of the channel establishes an outward current that causes cells hyperpolarization, inhibiting excitability and consequently pain transmission.^[^ ^73^ ^]^ Loss of function mutations of the *KCNK18* gene are involved in congenital migraine with aura onset.^[^ ^74^ ^]^ FFA and a series of derivatives were shown to potentiate hTRESK currents in transfected HEK cells, probably by stabilizing the open state of TRESK.^[^ ^75^ ^]^

### 2.3 Inwardly Rectifying Potassium Channels

Inwardly rectifying potassium channels (Kir) allow K^+^ ions to move into the cell rather than out.^[^ ^76^ ^]^ Fifteen KCNJx genes are known to encode human Kir channels with different functions and endogenous modulators, and relatively poor pharmacology.

Fenamate modulation was observed on ATP‐sensitive potassium channels (or K_ATP_ channel). These channels are composed by four pore‐forming (Kir6.x) subunits and four regulatory sulfonylurea receptor (SURx) subunits and are expressed mainly in pancreas, brain, and muscles. They are inhibited by high intracellular ATP, thereby coupling intracellular metabolism to plasma membrane potential. In the pancreas, they play a critical role in insulin secretion and are targets of hypoglycemic drugs.^[^ ^77^ ^,^ ^78^ ^]^ It has been hypothesized that K_ATP_ channel channels may be directly involved in the onset of severe hypoglycemia that can occur with the use of NSAIDs, especially in concomitance with glucose‐lowering drugs like sulfonylureas.^[^ ^79^ ^]^ To better characterize this phenomenon, NSAIDs were tested on a beta‐pancreatic cell line, INS‐1 cells. Through the inhibition of K_ATP_ channel, MFA‐induced cell depolarization (EC_50_ = ≈25 µM) and a consequent Ca^2+^ influx through voltage‐gated Ca^2+^ channel, causing insulin release, only in low glucose condition. Interestingly, while sulfonylureas bind the intracellular side of the channel, MFA likely binds the extracellular side, suggesting a possible synergistic activity that may enhance propensity for hypoglycemia.

Surprisingly, an opposite activity of MFA was reported in smooth muscle cells from pig proximal urethra.^[^ ^57^ ^]^ Application of 300 µM MFA hyperpolarized the cell membrane in current‐clamp experiments and activated K^+^ currents at −50 mV in whole cells and cell‐attached patches, both effects being prevented by the K_ATP_ channel inhibitor glibenclamide.

Further experiments would be useful to address this apparent discrepancy.

## 3 Sodium Channels and Fenamates

Sodium channels are widely expressed in mammalian cells and include voltage‐gated sodium channels (VGSC) and non‐VGSC like epithelial sodium channels (EnaC) that mediates sodium transport in epithelia. VGSC are involved in the genesis and propagation of action potential in excitable cells, like neurons, myocytes, and endocrine cells.^[^ ^80^ ^,^ ^81^ ^]^ They consist of a multimeric complex, made up of an *α* subunit of 260 kDa and one or more smaller auxiliary *β* subunits of 33–36 kDa (*β*1, *β*2, *β*3).^[^ ^82^ ^]^ Nine isoforms of the VGSC channel have been described in humans (Nav1.1‐Nav1.9)^[^ ^83^ ^]^ and their dysfunction are linked to a series of neurological, cardiac and muscle diseases, cancer, immune system disorders, and pain.^[^ ^5^ ^,^ ^84^, ^85^ ^–^ ^86^ ^]^

**Figure 3:**
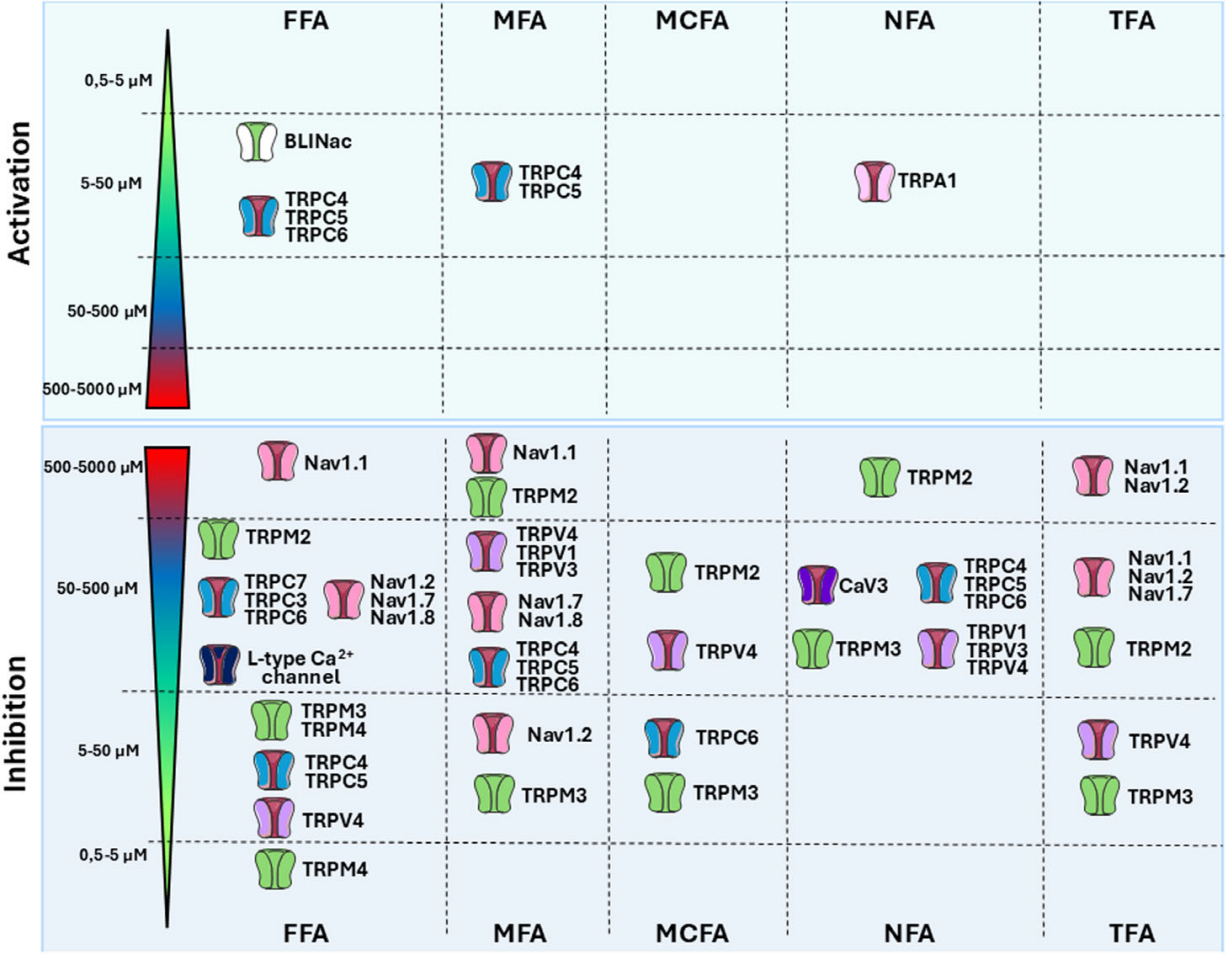
Effects of fenamates on sodium, calcium, and non‐selective cationic channels. Colored scale reported the estimated range of IC50 or concentration tested for each fenamates. These inhibitory or activation values are not always fully comparable since they have been evaluated on different systems (heterologous or native systems, in vitro or ex vivo) by considering different effects (current amplitude, voltage dependence). Dual effects of MFA and FFA are reported on TRPC4, TRPC5, and TRPC6 channels. Detailed information, abbreviations, and references are provided within the main text.

A few studies are available about fenamates activity on sodium channels (**Figure** **3**). A potential role of FFA as antiepileptic drug was proposed after observing that it could inhibit voltage‐gated sodium currents in hippocampal pyramidal neurons in a concentration‐dependent manner (IC_50_ = ≈190 µM).^[^ ^87^ ^]^ FFA mainly favored channel inactivation, shifting the curve to more hyperpolarized potential and slowing down the recovery phase. VGSC inhibition likely accounted for the decrease of pyramidal cell firing by FFA. However, a recent study suggested that the anti‐seizures effects of fenamates might rely more on GABA_A_ receptor stimulation rather than sodium channel inhibition.^[^ ^88^ ^]^

FFA and diclofenac were shown to inhibit sodium currents in rat DRG neurons.^[^ ^89^ ^]^ Interestingly, FFA (100 µM) was more active on tetrodotoxin (TTX)‐sensitive sodium currents, likely carried by Nav1.7, than TTX‐resistant currents, likely carried by Nav1.8. Such distinct effects were confirmed in CHO cells transfected with human Nav1.7 or Nav1.8 channels.^[^ ^90^ ^]^ In addition, inhibitory effects of MFA and TFA were also reported, the former being quite equipotent to FFA on hNav1.7, while the later was less potent. Such results suggest that inhibition of Nav1.7 might contribute to the analgesic effects of fenamates, beside cyclooxygenase inhibition.

Fenamates also inhibited heterologously expressed human Nav1.2 channels, while only small effects were found on hNav1.1^[^ ^91^ ^]^ MFA (IC_50_ = ≈30 µM) was more potent than FFA and TFA. A negative shift of activation and fast inactivation of Nav1.2 was reported as well as a slowing down of recovery from inactivation. In SH‐SY5Y, fenamates exerted a neuroprotective effect against glutamate‐induced apoptosis likely through inhibition of sodium channels and NMDA receptor‐evoked currents.

Besides VGSC, a few studies observed that FFA activated a sodium conductance in ventricular cardiomyocytes and the eligible channel responsible for this current was the brain liver intestine Na^+^ channel (BLINaC), an ion channel belonging to the DEG/ENaC gene family.^[^ ^92^ ^,^ ^93^ ^]^

## 4 Calcium Channels and Fenamates

Calcium channels play a central role in cell biology. The calcium ions are implicated in membrane potential modulation and constitute a pivotal intracellular signal acting as a second messenger in many pathways. Fenamates are well‐known modulator of cellular calcium concentration (Figure 3) and this effect was first observed in 1995 by Kankaanranta and colleagues.^[^ ^94^ ^,^ ^95^ ^]^ They noticed that FFA and TOLF inhibit the calcium influx evoked by the calcium ionophore A23187 and the chemotactic peptide fMLP, thereby negatively affecting the function of human leukocytes and neutrophils. Other studies have investigated the effects of fenamates on voltage‐gated calcium channels (Cav). Balderas and collaborators analyzed the effect of NFA on Ca_V_3 channels in mouse spermatogenic cells and in HEK cells expressing Ca_V_3 isoforms (Ca_V_3.1, Ca_V_3.2 and Ca_V_3.3).^[^ ^96^ ^]^ They demonstrated that NFA inhibited T‐type current with an IC_50_ of 74 µM in a voltage‐independent manner in mouse spermatogenic cells, with a similar effect on heterologously expressed Ca_V_3.2, while a small voltage dependence was observed in Ca_V_3.1 and Ca_V_3.3 inhibition. The strongest affinity of NFA was for Cav3.1. Another study showed that FFA blocked L‐type Ca^2+^ channels (IC_50_ = ≈100 µM) in carotid artery endothelial cells derived from spontaneously hypertensive stroke prone rats, with a consequent inhibition of smooth muscle tone.^[^ ^97^ ^]^ A light inhibitory effect of NFA was also observed on voltage‐gated calcium channel expressed in chemoreceptor cells of the rat carotid body, involved in cardiorespiratory regulation.^[^ ^98^ ^]^

Additionally, fenamates may increase intracellular Ca^2+^ concentration through a mechanism independent from cyclooxygenase inhibition that involves calcium release from mitochondria that, in turn, may modulate the activity of various channels, including the ClC‐1 chloride channels in rat skeletal muscle fibers.^[^ ^99^ ^]^

## 5 Non‐Selective Cation Channels and Fenamates

Non‐selective cation channels (NSCC) are a heterogeneous group of channels that discriminate according only to cation charge. This group includes the transient receptor potential (TRP) channels, ligand‐gated NSC channels, connexins, and cyclic nucleotide‐gated channels. A pioneering study reported the inhibition of NSC currents in rat exocrine pancreas by FFA, MFA, and NFA but the molecular correlate was not known at this time.^[^ ^100^ ^]^ Since then, many NSCC have been shown modulated by fenamates (Figure 3).

### 5.1 Transient Receptor Potential Channels (TRP)

TRP channels are weakly voltage‐sensitive tetrameric pores that allow the influx of Na^+^ and Ca^2+^ into the cells. In humans, 27 TRP channels belonging to six families (TRPA, TRPC, TRPM, TRPML, TRPP, and TRPV), are involved in a plethora of cell function and diseases.^[^ ^101^ ^]^ Many TRP channels are modulated by fenamates.

In humans, TRPA1 is the unique member of TRPA family. TRPA1 is expressed in neurons and activated by external irritants and endogenous algogenic agents, being involved in nociception, itching, cough as well as in acute inflammatory pain and cardiovascular pathophysiology.^[^ ^102^ ^,^ ^103^ ^]^

In *Xenopus* oocytes expressing rat TRPA1, application of FFA enhanced Ca^2+^ currents in a dose‐dependent manner, with IC_50_ of 78 and 147 µM at + 100 and −100 mV, respectively.^[^ ^104^ ^]^ NFA was about three times less potent than FFA. Fenamates (FFA, NFA, and MFA) exerted synergistic effects with other TRPA1 agonists. Similar effects were obtained with a series of NSAIDS, including indomethacin, or flurbiprofen. Accordingly, diclofenac, ketorolac and xefocam reduced thermal and mechanical hyperalgesia induced by TRPA1 activation by agonistic molecules (e.g., cinnamaldehyde and allyl isothiocyanate) in animal models. A similar effect was also observed after stimulation of TRPV1 by capsaicin.^[^ ^105^ ^]^ Furthermore, it was found that the analgesic effect of ibuprofen was partly mediated by TRPA1 modulation through its metabolite acyl‐glucuronide.^[^ ^106^ ^]^ By contrast, ketorolac and naproxen did not show an effect on TRP channels, indicating that the modulatory effect on TRPA1 is restricted to certain NSAIDs including fenamates.^[^ ^104^ ^]^ Further studies are warranted to better understand the role played by TRPA1 in fenamate analgesic activity.

In contrast to TRPA1, fenamates are mostly inhibitors of TRPC channels, which behave as store‐operated or receptor‐operated channels. FFA (100 µM) was shown to inhibit murine TRPC3 and TRPC7 channels expressed in HEK cells.^[^ ^107^ ^]^ FFA inhibited TRPC3 channels in outside‐out patches from rabbit ear or coronary artery myocytes with an IC_50_ of 2–7 µM, suggesting a direct effect on the channel.^[^ ^108^ ^,^ ^109^ ^]^ Inhibition of TRPC3 channels in GABAergic neurons of Subtantia Nigra by 100 µM FFA was shown to reduce tonically active inward Na^+^ currents, inducing membrane hyperpolarization and dampening neuron firing.^[^ ^110^ ^]^ Further studies suggested a role for TRPC3 in neuron firing and its sensibility to FFA inhibition [see for instance].^[^ ^111^ ^,^ ^112^ ^]^ It should be mentioned however that interpretation of pharmacological data in native cells can be challenged by the co‐expression of various TRPC subtypes. A blocking activity of FFA was also recorded on human TRPC4 and TRPC5 channels expressed in HEK cells, with IC_50_ of 55 and 37 µM, respectively.^[^ ^18^ ^]^ MFA, NFA and diclofenac were also inhibitors but at higher concentrations (IC_50_ > 80 µM). These effects were not reproduced by other COX inhibitors, suggesting again a direct interaction of fenamates with the channels. Interestingly, a series of FFA analogs with various substitutions of the trifluoromethyl group were all less potent than the parent compound, and additional substituents on the phenylamino ring swifts to a TRPC4 activator. Noticeably, after a prolonged perfusion (> 5 min) of FFA and MFA (but NFA or diclofenac), a delayed activation of TRPC4 and TRPC5 channels was observed due to an increased release of cytosolic Ca^2+^ from mitochondria. Such observation further highlights the possible difficulties in pharmacological data interpretation.

The TRPC6 channel, which is an *α*‐adrenoreceptor‐activated and Ca^2+^‐permeable NSC channel, might be distinguished form other TRPC channels by its activation by FFA. Activation of rodent and rabbit TRPC6 by FFA was reported in HEK cells and various cell preparations.^[^ ^107^ ^,^ ^113^, ^114^ ^–^ ^115^ ^]^ A direct activation of hTRPC6 by FFA, independent from COX inhibition, was further reported.^[^ ^116^ ^]^ Although these observations were challenged in a few studies suggesting either lack of effect or inhibition of TRPC6 channels,^[^ ^117^, ^118^ ^–^ ^119^ ^]^ FFA is widely considered as a TRPC6 activator and used as such in functional studies.

Fenamates are also more or less selective inhibitors of TRPM channels. TRPM2 is a temperature‐sensitive NSC channel playing an important role in coupling oxidative stress to Ca^2+^ influx.^[^ ^120^ ^]^ It is expressed in a wide number of tissues, including the brain, vascular smooth muscle and endothelial cells, immune and endocrine cells. In rat insulinoma cell line and in HEK293 cells expressing human TRPM2, FFA exerted a complete inhibitory activity at different concentrations (50–1000 µM).^[^ ^121^ ^]^ After FFA washout, only 10–15% of current was restored, suggesting an irreversible effect of the drug on this channel. Furthermore, lowering pH accelerated TRMP2 inhibition by FFA, demonstrating that extracellular pH affected FFA activity. The FFA‐mediated inhibition of TRPM2 expressed in pancreatic islets induced a significant reduction of glucose‐induced insulin release, suggesting TRPM2 involvement in insulin secretion pathway.^[^ ^122^ ^]^ The inhibitory action of FFA on TRPM2 was confirmed in several other studies.^[^ ^123^ ^,^ ^124^ ^]^ TRPM2 was also inhibited by MCFA and TFA with about two‐fold greater potency compared to FFA, whereas NFA and MFA had reduced potency.^[^ ^119^ ^,^ ^125^, ^126^ ^–^ ^127^ ^]^

TRPM3 is a NSC channel permeable to Ca^2+^, expressed in many tissues, activated by voltage, heat, chemicals, and endogenous ligands such as neurosteroids, and inhibited by G_*βγ*_ subunit.^[^ ^128^ ^]^ Gain of function mutations of TRPM3 have been linked to developmental and epileptic encephalopathies. MFA was reported as a relatively selective inhibitor of TRPM3 expressed in HEK cells, with an IC_50_ of 7 µM.^[^ ^119^ ^]^ TFA and MCFA were slightly less potent than MFA, followed by FFA then NFA. TRPM3 inhibition by MFA was confirmed in different cellular models, like retinal ganglion cells and insulin‐secreting INS‐1E cells.^[^ ^119^ ^]^ In Purkinje glutamatergic synapsis, TRPM3 inhibition by MFA resulted in the suppression of glutamatergic transmission in developing synapsis.^[^ ^129^ ^]^ Due to its blocking activity on TRPM3, MFA may have anti‐tumoral properties. Indeed, it was shown that TRPM3 promotes cancer cells proliferation in different tumors, like clear cell renal cell carcinoma (ccRCC), glioblastoma, and choroid plexus papilloma, probably through activation of oncogenic pro‐survival autophagy pathway.^[^ ^130^ ^,^ ^131^ ^]^ MFA treatment on ccRCC xenograft tumors reduced tumor growth and achieved tumor regression. Furthermore, MFA affected TRPM3 gene transcription and reduced the expression of TRPM3 channel in tumor cells.^[^ ^131^ ^]^

Compared to other TRP channels, both TRPM4 and TRPM5 present the distinctive properties of not being permeable to Ca^2+^, while being activated by intracellular Ca^2+^ overload.^[^ ^132^ ^]^ TRPM4 was ten fold more sensitive than TRPM5 to FFA inhibition in inside‐out patches from transfected HEK cells (IC_50_ = 2.8 µM versus 24.5 µM, respectively).^[^ ^133^ ^]^ Consequently, FFA has been as a pharmacological tool to characterize TRPM4 channels in native cells [see for instance].^[^ ^134^ ^]^ Recently, a thallium assay‐based screening of 2560 compounds revealed MCFA as a novel potent TRPM4 antagonist [IC_50_ = 3.4 µM], able to inhibit the Ca^2+^ overload‐induced background current in ventricular cardiomyocytes and to suppress catecholaminergic polymorphic ventricular tachycardia‐associated arrhythmias in mice.^[^ ^135^ ^]^

The transient receptor potential vanilloid (TRPV) channels are implicated in somatic pain, expressed in dorsal root ganglia, central nervous system, liver, and kidney. FFA 100 µM (as well as NFA and MFA) was reported as an inhibitor of murine TRPV1 and TRPV3 channels in Xenopus oocytes.^[^ ^136^ ^]^ In HEK cells expressing TRPV4, TFA was the most potent inhibitor among fenamates (IC_50_ = 24 µM), followed by FFA and MCFA, then NFA and the quite inactive MFA.^[^ ^119^ ^]^ Of note, dopamine amide substituted MFA and FFA exerted agonistic effects on hTRPV1 expressed in HEK cells with EC_50_ as low as 15–35 Nm.^[^ ^137^ ^]^

Hence, FFA, MFA, and NFA showed different modulatory activities on TRP channel, with both blocking or enhancing effect, depending on specific channels.

### 5.2 Ligand‐Gated NSC Channels

N‐methyl‐D‐aspartate (NMDA) glutamate receptor and neuronal nicotinic acetylcholine receptors (nAChRs) are ligand‐gated NSC channels that may be modulated by fenamates.

The NMDA glutamate receptor is found in spinal cord neuron and enables Ca^2+^ flux that consequently activates a Ca^2+^‐dependent NSC channel (NSC_Ca_). A first study reported that NMDA currents in cultured spinal cord neurons are inhibited by high FFA and NFA concentrations (IC_50_ = ≈350 µM).^[^ ^138^ ^]^ Then, it was suggested that FFA, MFA and MCFA (1 mM) may protect neurons from ischemia excitotoxic damage through inhibition of NMDA receptors.^[^ ^139^ ^]^ NMDA receptors are also involved in epilepsy and 100 µM FFA was able to block epileptiform activity in the hippocampus. Anyways, because FFA can inhibit NSC_Ca_ as well, it was not clear if this phenomenon could be strictly attributed to FFA inhibition of NMDA receptor.^[^ ^140^ ^]^ In addition, lack of effect of 100 µM MFA on NMDA receptors in hippocampal neurons was recently reported, while fenamates dose‐dependently potentiated GABA‐evoked currents, supporting a role for GABA_A_ receptor modulation in anti‐epileptic effects of fenamates.^[^ ^141^ ^]^

Neuronal nicotinic acetylcholine receptors (nAChRs) are NSC pentameric channels with high permeability to Ca^2+^. FFA and NFA exerted either agonistic or antagonistic effect on nACHRs expressed in *Xenopus* oocytes according to their subunits composition.^[^ ^142^ ^]^ Indeed, both fenamates inhibited inward currents of *α*3*β*2 nAChR (IC_50_ = 90 and 260 µM for FFA and NFA, respectively) or increased *α*3*β*4 nAChR currents (EC_50_ = 30 µM for NFA, and FFA more efficient at any tested concentration). The authors claimed that the fenamate effects were independent from chloride channels and probably caused by direct action on nAChRs. However, a successive study also suggested that nAChRs activation in dopamine neurons might subsequently activate a Ca^2+^‐activated cationic current that was inhibited by FFA.^[^ ^143^ ^]^ Thus, further studies are warranted to better define the modulation of nAChrs by fenamates.

### 5.3 Connexins

Connexin‐based gap junctions connect adjacent cells electrically and are formed by two hemichannels, each composed of six connexin subunits and permeable to cations, anions and some signaling molecules. There are 21 different isotypes of these proteins in the human genome and 20 isotypes in the mouse genome,^[^ ^144^ ^]^ of which 11 expressed in the brain.

Fenamates were first identified as gap junction connexin blockers in electrically coupled fibroblasts of rat kidney or SK‐HEP‐1 cells overexpressing the connexin 43 (Cx43).^[^ ^11^ ^,^ ^145^ ^]^ The drugs inhibited both electrical and dye cell coupling with IC_50_ of 25 µM (MCFA) and 40 µM (FFA); NFA had intermediate potency. The effects were partly reversible. In *Xenopus laevis* oocytes, both FFA and NFA inhibited mouse Cx50 hemichannels with IC_50_ of 3 and 11 µM, respectively.^[^ ^146^ ^]^ NFA acted only from the outside by reducing Cx50 open probability. Interestingly, mouse Cx46 hemichannels were inhibited by FFA but were insensitive to NFA. In addition, FFA was shown to inhibit many other connexins (Cx23, 32, 40, 43, 46, 50) with IC_50_ of 20‐60 µM in transfected N2A neuroblastoma cells.^[^ ^147^ ^]^ The inhibition of cell coupling and/or connexin hemichannels by fenamates has been confirmed in a myriad of cell preparations [see for instance].^[^ ^140^ ^,^ ^148^ ^,^ ^149^ ^]^

### 5.4 Cyclic Nucleotide‐Gated Channels

Cyclic nucleotide‐gated channels encompass non selective cation channels activated by cGMP or cAMP. The hyperpolarization‐activated cyclic nucleotide‐gated cation 2 (HCN2) channel is responsible for cardiac and brain native pacemaker activity generation. NFA was shown to inhibit currents carried by HCN2 expressed in *Xenopus* oocytes, through slowing of activation/deactivation and shift of activation voltage‐dependence towards more negative potential.^[^ ^150^ ^]^ The authors suggested direct action of NFA with the S4 voltage sensor domain in closed state channels resulting in pore obstruction. It should be noted that these effects were observed at rather high NFA concentrations, with an EC_50_ > 500 µM on activation voltage dependence.

## 6 Conclusion

To date, many studies have shown that fenamates have a pharmacological activity independent from COX inhibition and from their conventional anti‐inflammatory and analgesic effects suggesting the existence of alternative targets with pleiotropic and potential therapeutic activity.

Among these, ion channels represent attractive targets that suggest the possibility of fenamates repurposing in ion‐channels‐related diseases.

Notably, fenamates have been shown to interact with a wide range of channel types, including both voltage‐gated and ligand‐gated channels. Although their activating or inhibiting effects on these proteins are well described, the underlying mechanisms remain largely unknown. Because fenamates targets numerous ion channels with different structures, biophysics, and regulatory properties, the underlying mechanisms might be different from one to the other. For instance, experiments performed on the calcium‐activated potassium channel KCa4.2 revealed that the introduction of a point mutation (A278R) in the sixth transmembrane segment adjacent to the pore significantly increased the efficacy of FFA, suggesting an involvement of this region in FFA binding.^[^ ^67^ ^,^ ^151^ ^]^ This binding site may be different in non‐selective cation channels, as in Cx50, where it may be a modulatory site comprised within the membrane but not in the pore.^[^ ^147^ ^]^ These findings underscore the idea that fenamates exert their effects through channel‐specific mechanisms, likely driven by the structural heterogeneity of their targets and the amphipathic nature of the compounds themselves. In this review, we have highlighted that many preclinical and clinical studies demonstrated the ability of fenamates to reduce neuropathic pain, cell proliferation and apoptosis as well as their anti‐seizures and anti‐arrthymic effects. Among these emerging roles, particular attention has been given to their effects in neurodegenerative disorders. Fenamates, such as MFA and FFA, have demonstrated neuroprotective properties through multiple mechanisms. These drugs are able to inhibit the NLRP3 inflammasome by blocking volume‐regulated anion channels (VRACs) in macrophages, thereby reducing neuroinflammation. In rodent models of Alzheimer's disease, treatment with MFA has been shown to decrease microglial activation and IL‐1*β* expression and improved cognitive function, highlighting the potential for repurposing fenamates as therapeutic agents in neurodegenerative conditions.^[^ ^152^ ^,^ ^153^ ^]^

Moreover, growing evidence suggests that fenamates exert anti‐cancer effects by modulating key pathways involved in cell proliferation, apoptosis, and gene expression. They have been shown to inhibit tumor cell growth, promote apoptosis, and interfere with signaling pathways involved in tumor progression such as Wnt/*β*‐catenin, NF‐*κ*B and p38 MAPK in various cancer models, suggesting their potential as adjunctive agents in cancer therapy.^[^ ^11^ ^]^

Accordingly, different clinical trials are currently investigating the efficacy of fenamates, alone or in combination with other drugs, for the treatment of neurodegenerative syndromes,^[^ ^152^ ^]^ brain metastasis (NCT02429570) and glioblastoma (EudraCT 2021‐000708−39) in order to obtain better patients’ outcome.

Nevertheless, an unsolved issue of repurposing fenamates involves their selectivity on specific ion channel (e.g., sodium, potassium, or calcium channels) to prevent potential off‐target effects due to the modulation of multiple ion channel subtypes. In addition, as many channelopaties are chronic diseases, another important challenge of fenamates repurposing is the management of their adverse effects. Indeed, NSAIDs may lead to gastrointestinal, cardiovascular, and renal complications, in particular, after their prolonged treatment. Therefore, in order to produce more effective and selective drugs able to target specific ion channels with fewer off‐target effects, molecular docking and structure‐based research may offer important insights into fenamates and ion channel interactions. Accordingly, the identification of fenamate derivatives that can selectively modulate specific cation ion channels (sodium, potassium, calcium or, in general, cationic channels) will provide novel drugs that may be applied for the treatment of different conditions, including epilepsy, chronic pain, and cardiac arrhythmias.

Furthermore, fenamates may also be used in combination strategies with other ion channel modulators and/or drugs with a different mechanism of action leading to additional therapeutic benefits, potentially providing synergistic effects in patients affected by complex genetic or acquired channelopathies. In addition, the use of combination therapies may allow clinicians to adjust treatment regimens based on specific patient profiles, improving both efficacy and safety providing a more tailored and effective approach for personalized medicine.

Overall, the repurpose of fenamates, as well as the development of new derivatives, should significantly expand their therapeutic applications. Hence, pharmacological research on fenamates through the knowledge of chemistry, genetics, pathophysiology, and clinical medicine should highlight the therapeutic potential of fenamates harnessing their benefits in clinical settings. These advances may offer novel perspectives for patients affected by a wide range of ion channel‐related disorders, potentially improving quality of life and clinical outcomes.

## Conflicts of Interest

The authors declare no conflict of interest.

## Author Contributions

**Paola Laghetti**, **Concetta Altamura**, **Simone Dell’Atti:** conceptualization, literature review, writing‐original draft. **Jean‐François Desaphy**, **Ilaria Saltarella:** conceptualization, review and editing. All authors have read and agreed to the published version of the manuscript.
